# Proteomic analysis of acidic chaperones, and stress proteins in extreme halophile *Halobacterium *NRC-1: a comparative proteomic approach to study heat shock response

**DOI:** 10.1186/1477-5956-4-6

**Published:** 2006-04-19

**Authors:** Hem D Shukla

**Affiliations:** 1 Center of Marine Biotechnology, University of Maryland Biotechnology Institute, Baltimore, MD 21202, USA

## Abstract

**Background:**

*Halobacterium *sp. NRC-1 is an extremely halophilic archaeon and has adapted to optimal growth under conditions of extremely high salinity. Its proteome is highly acidic with a median pI of 4.9, a unique characteristic which helps the organism to adapt high saline environment. In the natural growth environment, *Halobacterium *NRC-1 encounters a number of stressful conditions including high temperature and intense solar radiation, oxidative and cold stress. Heat shock proteins and chaperones play indispensable roles in an organism's survival under many stress conditions. The aim of this study was to develop an improved method of 2-D gel electrophoresis with enhanced resolution of the acidic proteome, and to identify proteins with diverse cellular functions using in-gel digestion and LC-MS/MS and MALDI-TOF approach.

**Results:**

A modified 2-D gel electrophoretic procedure, employing IPG strips in the range of pH 3–6, enabled improved separation of acidic proteins relative to previous techniques. Combining experimental data from 2-D gel electrophoresis with available genomic information, allowed the identification of at least 30 cellular proteins involved in many cellular functions: stress response and protein folding (CctB, PpiA, DpsA, and MsrA), DNA replication and repair (DNA polymerase A α subunit, Orc4/CDC6, and UvrC), transcriptional regulation (Trh5 and ElfA), translation (ribosomal proteins Rps27ae and Rphs6 of the 30 S ribosomal subunit; Rpl31eand Rpl18e of the 50 S ribosomal subunit), transport (YufN), chemotaxis (CheC2), and housekeeping (ThiC, ThiD, FumC, ImD2, GapB, TpiA, and PurE). In addition, four gene products with undetermined function were also identified: Vng1807H, Vng0683C, Vng1300H, and Vng6254. To study the heat shock response of *Halobacterium *NRC-1, growth conditions for heat shock were determined and the proteomic profiles under normal (42°C), and heat shock (49°C) conditions, were compared. Using a differential proteomic approach in combination with available genomic information, bioinformatic analysis revealed five putative heat shock proteins that were upregulated in cells subjected to heat stress at 49°C, namely DnaJ, GrpE, sHsp-1, Hsp-5 and sHsp-2.

**Conclusion:**

The modified 2-D gel electrophoresis markedly enhanced the resolution of the extremely acidic proteome of *Halobacterium *NRC-1. Constitutive expression of stress proteins and chaperones help the organism to adapt and survive under extreme salinity and other stress conditions. The upregulated expression pattern of putative chaperones DnaJ, GrpE, sHsp-1, Hsp-5 and sHsp-2 under elevated temperature clearly suggests that *Halobacterium *NRC-1 has a sophisticated defense mechanism to survive in extreme environments.

## Background

*Halobacterium *sp. NRC-1 inhabits an extreme and dynamic environment which presents a significant challenge to its survival. The organism has the capability to adapt to 4.5 M of salt and exhibit growth up to about 50°C, albeit at reduced rate. It responds to a wide range of environmental perturbations including intense solar radiation, high temperature, cold, high salinity and oxidative stress [1-3]. The cells can grow using both aerobic and anaerobic respiration and have anaerobic fermentation and phototrophic capabilities [4]. The organism is genetically tractable with a wide variety of genetic tools, including cloning vectors, selectable markers, and a facile gene knockout system [5]. These facts, together with the availability of its complete genome sequence [4], make *Halobacterium *sp. NRC-1 an ideal system for studying responses to environmental perturbations.

The genome sequences of *Halobacterium *sp. NRC-1 have shown the presence of three replicons: a 2 Mbp chromosome, and two minichromosomes of 365 kbp (pNRC200), and 191 kbp (pNRC100), encoding 2630 predicted proteins [4]. Bioinformatic analyses of the NRC-1 genome have shown the predicted proteome to be extremely acidic, with a median pI of only 4.9 [2]. Preliminary experimental proteomic analysis of *Halobacterium *NRC-1 and related halophiles has exhibited the feasibility of both two-dimensional gel electrophoretic (2-DE) analysis [6,7], liquid chromatography coupled tandem mass spectrometry (LC-MS/MS), and MALDI-TOF analysis [7,8]. Furthermore, mass spectrometric analysis of purple membrane preparations from *Halobacterium *has also been reported [9]. However, these studies have not extensively analyzed the changes in abundance of individual proteins or patterns under differential growth conditions.

Despite the recent advances in proteomic research, and improved 2-DE systems, the analysis of the *Halobacterium *NRC-1 proteome is a considerable challenge [7]. Because of its extreme acidity, and relatively high hydrophobicity, proteins tend to precipitate at their isoelectric point during IEF analysis [10,11]. Moreover, presence of salt in the sample and excess DTT at acidic pH leads to streaking and skewed results. These problems have previously been approached using low molecular weight cut-off columns, ultrazoom immobilized pH gradient strips, and combinations of IPGphor/Multiphor systems [7,12]. However, streaking at the high molecular weight region of the 2-D gels still poses considerable challenge in obtaining reproducible results, which are essential for comparative study and global protein analysis in extreme halophiles.

Heat shock response is an important homeostatic mechanism that enables cells to survive a variety of environmental stresses [13]. Some heat shock proteins are constitutively expressed in extremophiles under normal growth conditions, suggesting that they have evolved spontaneous adaptation to extreme environmental conditions [14]. These heat shock proteins function in multi-protein complexes as molecular chaperones and assist in the proper protein folding of stress damaged proteins, and stabilization of other cellular proteins [15-18].

*Halobacterium *NRC-1 is challenged by hostile conditions in its natural environment including intense solar radiation, high temperature, high salinity and oxidative stress. The complete genome sequence of *Halobacterium *NRC-1 has revealed the presence of 13 heat shock genes which belong to five major families of Hsps including alpha and beta thermosomes, DnaK, DnaJ, GrpE, DpsA, MsrA and sHsps [4]. Among them, *Halobacterium *NRC-1 genome contains 3 copies of small heat shock proteins (sHsps) whereas, other archaeal genomes contain only one or two copies each. Small heat shock proteins are less than 25 kDa in size and ubiquitous in all types of organisms, including archaea, bacteria, and eukarya [19].

In present study, a modified 2-DE procedure is employed to improve resolution of acidic proteins. Using the modified procedure, 30 abundantly expressed proteins were identified following LC-MS/MS and MALDI-TOF analyses. Further, by employing a differential protein expression analyses approach, combined with bioinformatics analyses, an attempt has been made to identify putative heat shock proteins and chaperones up regulated during temperature stress in this model extremophile.

## Result and discussion

### Optimization of 2-D gel electrophoresis for resolution of acidic proteins

**Figure 1:**
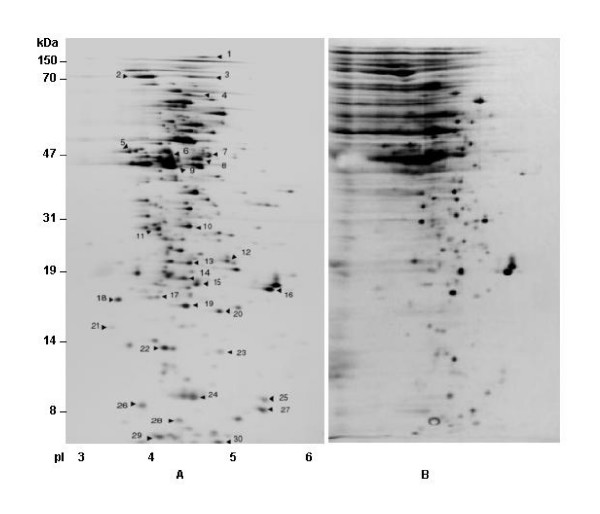
**A.** Typical image of 2-D gel electrophoresis analysis of intracellular proteins of *Halobacterium NRC-1* (pI 3-6) by modified procedure, cells were grown under normal growth conditions. **B.** 2-D gel analysis of intracellular acidic proteins of *Halobacterium NRC-1* by unmodified procedure.

Halobacterial proteins are extremely acidic and have highly negatively charged surfaces, which are thought to enhance solubility and maintain function at high salinity [20]. In order to improve resolution and minimize streaking of proteins in the acidic range, three modifications were introduced to a recently published procedure [7]: (1), the sample was extensively dialyzed using 3 kDa cutoff dialysis bag with at least 4 buffer changes; (2), 2.0 % Tween-20, a non-ionic detergent, was used in the rehydration buffer in place of NP-40; and (3), the rehydration step was carried out at 50 volts for 16 hrs at 22°C. Apparently, extended rehydration of IPGready strips at low voltage prevented the usual precipitation observed at acidic pH. These modifications effectively removed the salt and concomitantly allowed the acidic proteins to focus more efficiently at the acidic range of the IPG strip. As shown in Fig. 1, proteins were well resolved in the acidic range and very limited streaking was observed at higher MW range, unlike earlier reports [6,7,21,22]. However, acidic proteins which were resolved through unmodified 2-D gel procedure resulted in horizontal and vertical streaking at higher MW range. (Fig. 1B).

**Figure 2:**
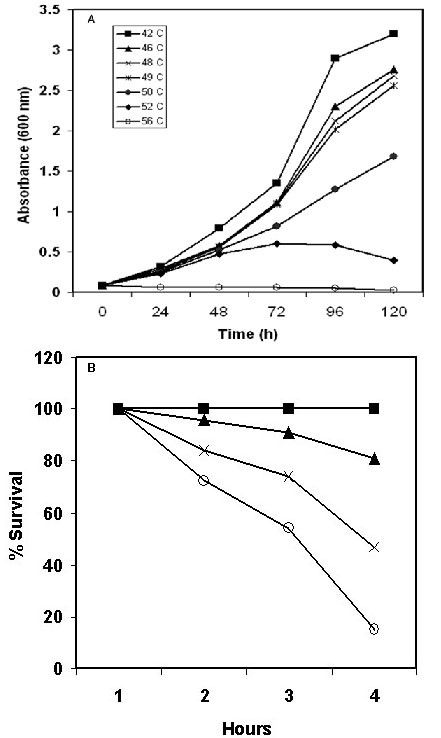
**A.** Growth curves of *Halobacterium* NRC-1 at elevated temperatures 42 (squares), 46 (triangles), 48 (cross), 49 (asterisks), 50 (dark circles), 52 (diamonds), and 56 °C. (open circles). **B.** Survival of *Halobacterium* NRC-1 at sublethal (49 °C- triangles), Pretreatment of cells at 49 °C for 1 hour, then shifted at 56 °C (cross), and directly at lethal (56 °C- open circles) temperature. Control was grown at 42 °C (dark squares).

By employing the modified protocol for analytical 2-DE of acidic proteins, thirty abundantly expressed proteins were identified under standard laboratory growth conditions at 42°C [5]. The data presented in Fig. 1 represent the reference map, which was generated using IPG ready strips of the pH range 3–6. In the present approach, a high degree of reproducibility was observed between gels, and replicate gel images were averaged and analyzed. Analyses of resulting gels were performed using ImageMaster software and approximately 411 protein spots were detected in the range of 10–150 kDa with pI's in the range of 3 to 6. The average pI of the expressed proteome was found to be 4.42, which is in close agreement with the predicted proteome derived from genome sequence [4]. Multiple experiments revealed that using the Image Master 2-D Software, highly reproducible data were obtained which was corroborated by earlier reports [23]. In present study, experimental pI and molecular weights were closely matched with the predicted values obtained from genome sequence. In additional experiments, few if any proteins in the neutral and basic range could be identified (data not shown), although 390 polypeptides are predicted in the *Halobacterium *NRC-1 genome sequence in the pI range of 7–11, similar problems were also encountered in earlier reports [24-26].

### Identification and analysis of cellular chaperones, and abundantly expressed gene products under 4.5 M of salt

Based on the protein profiles of 411 acidic proteins in reference 2-D maps, 30 abundantly expressed proteins representing a wide range of pI and MW were selected for identification. Spots were excised, digested with trypsin, and the resulting peptides were analyzed by MALDI-TOF and LC-MS/MS analysis. The results presented in Fig. 1 and Table 1 show that the abundantly expressed proteins that could be identified are involved in a wide range of cellular processes. In this novel approach the peptide masses obtained from MALDI-TOF were searched against MASCOT database and top hits were precisely matched with the pI and molecular weights of predicted proteins from genome sequence, and data obtained from 2-D gel electrophoretic analyses. Thus, only closely matched values were taken into consideration for identification of proteins. Interestingly, this approach could easily identify false positive despite the fact that the MASCOT scores of some false positives were found to be higher than the actual protein identified. However, the above stated approach discounted the probability of identifying false positives and allowed us to identify the actual candidates. Similarly, the MS/MS spectra were searched against NCBInr database using SEQUEST as described in materials and methods. Proteins identified by LC-MS/MS and MALDI-TOF analysis, with experimental and theoretical (M_r _and pI) values are presented in Table 1 with the ORF number, protein name, MS score and percent coverage. The bioinformatics analysis of identified proteins has shown that their pI and molecular weights match with the predicted values from genome sequence [4].

**Table 1:** List of proteins on the 2-DE map of *Halobacterium *NRC-1, identified byMALDI-TOF and LC-MS/MS

No.	ORF No.	Protein Name	MS/MASCOT Score	Mowse Score	% Coverage	Annotation	Putative kDa/pI	Calculated kDa/pI
1	2338	polA2*	15	39	13	DNA polymerase	150/4.38	148.9/4.39
2	2096	***cctB***	668		33	**Thermosome β**	70/4.12	69/4.1
3	2381	uvrC*	40	51	18	Excision nuclease chain C	63.7/4.8	63/4.89
4	6363	***Orc4/CDC6***	82		30	**Orc4/CDC6**	58/4.51	58/4.52
5	715	*thiC*	57		28	Thiamine biosynthesis protein	51/4.15	51.5/4.15
6	1601	gcvP2*	20	47	20	Glycine Dehydrogenase II	50/4.43	51/4.4
7	1356	*fumC*	378		27	Fumarate hydratase	50/4.4	50/4.38
8	2649	*eef1A*	158		18	Translation elongation factor α subunit	46/4.32	46.2/4.3
9	2606	*thiD*	83		21	Thiamine biosynthesis protein	45/4.2	47/4.17
10	95	*gapB*	220		13	Glyceraldehyde 3-PDH	35/4.26	35.5/4.2
11	903	yufN*	20	47	8	ABC transporter	33/4.02	33.2/4.02
12	683	vng0683C*	73	63	14	Hypothetical	28/4.69	28.9/4.6
13	2331	vng0309C*	56	46	12	Hypothetical	25/4.48	26.9/4.31
14	1027	*tpiA*	50		13	Triosephosphate isomerase	21/3.97	21.3/4.01
**15**	2443	***dpsA***	60		19	**Stress induced DNA binding protein**	20/4.14	20.4/4.16
16	890	*imD2*	196		25	Inosine-5'-monophosphate dehydrogenase	20/4.76	20.1/4.8
**17**	1914	ppiA*	20	45	11	**Peptidyl proryl isomerase**	20/4.02	21/4.1
18	633	*purE*	72		20	Phosphoribosylamino subunit	21/4	21.7/4.07
**19**	1180	***msrA***	71		38	**Peptide methionine sulfoxide red.**	19.4/4.2	21.1/4.18
20	1607	*cheC2*	76		12	Chemotaxis protein	21/4.23	21.7/4.2
21	2118	pyrE2*	25	51	22	Orotatephosphoribosyl transferase	18/4.11	19.2/4.13
22	626	maoC2*	27	52	22	Acyl Dehydratase	16.8/4.15	16.9/4.2
23	1807	*vng1807H*	55		38	Hypothetical	15/4.29	16/4.32
24	6254	vng6254*	21	42	12	Conserved Hypothetical	11.4/3.85	12.2/3.9
25	1137	rpl18E*	22	44	17	Ribosome assembly	12.5/5.6	12.1/5.62
26	1157	rphs6*	30	47	12	Ribosome assembly	12.7/3.79	12.5/3.81
27	2467	*rpl31E*	110		71	Ribosome assembly	10/4.52	10.2/4.4
28	1922	trh5*	32	54	26	**Lrp like regulator**	8.0/4.24	8.4/4.11
29	1300	vng1300H*	49	71	91	Hypothetical	6.5/3.91	8.0/4.0
30	2047	rps27aE*	30	49	10	Ribosome assembly	6/3.87	8.2/3.86

*Proteins identified by MALDI-TOF

### Protein folding and stress responses

CctB is a beta subunit of the thermosome, which belongs to group two chaperonins in archaea and is involved in various cellular functions during stress [27]. It has been reported to suppress aggregation of normal proteins under high salt [28]. It has been observed that under temperature stress, the thermosome spontaneously assembles into filaments, which suggested that they may play a structural role *in vivo *[29]. In addition, other functions have been suggested for group II chaperonins which help to adapt stressful conditions, including membrane stabilization [29]. The other chaperone identified under normal growth conditions was peptidyl-prolyl isomerase or rotamase (PpiA), which facilitates proper protein folding by increasing the rate of transition of proline residues between the *cis *and *trans *states [30]. The present approach also helped identify MsrA, a stress protein involved in cellular protection from oxidative stress damage [31]. MsrA has been reported to act against oxidative damage by inactivating oxygen radicals which have the tendency to damage DNA [32]. DpsA, another protein involved in protection of DNA damage from oxidative stress, [33], could also be identified. However, recent reports suggest that DpsA protects cells against multiple stresses during stationary phase [34]. The data obtained by combination of 2-D gel, LC-MS/MS and MALDI-TOF clearly indicate that that some of the chaperones and stress proteins are constitutively expressed in the cell. This might be due to the fact that *Halobacterium *NRC-1 inhabits an extreme environment of high salinity and other stresses [19] and, therefore, has evolved a unique and novel strategy for impromptu adaptation to extreme salinity and other stresses.

### DNA replication, transcription, translation and repair

The survival of *Halobacterium *NRC-1 under extreme environmental conditions always depends upon the successful repair and replication of genetic material. The proteomic analysis has shown the presence of polymerase A (α subunit) involved in DNA replication, and Orc4/CDC6 which plays an indispensable role in initiation of replication by binding to origin of replication. Findings also suggest that CDC6, which has AAA domain structure, is involved in the refolding of the denatured protein, protein turnover, and posttranslational modification under extreme salinity [35]. The identification of excision nuclease chain C (UvrC), a member of both UvrABC system and orthologous family COG0322, indicate that growing cells are exposed to high solar and UV radiation, and its expression successfully repairs the cellular DNA damage [36]. The presence of transcription regulation (Trh5, ElfA), translation [two 30S (Rps27ae, Rphs6), two 50 S (Rpl31e, Rpl18e) ribosomal proteins] gene products indicate that despite extreme stress conditions cell successfully maintain and regulate genetic information to its successful survival.

### Identification of transport, chemotaxis, and housekeeping gene products

The proteomic analysis has also shown the presence of YufN protein which is a member of the ABC transporter family embedded in halobacterial membranes containing the pfam 02608 domain. YufN is involved in the import of nutrients into cells or the release of toxic products into the surrounding medium and functions at the expense of ATP hydrolysis [37]. This class of proteins is the most important family of membrane transporters in *Halobacterium *NRC-1 genome.

CheC2 is a chemotaxis protein which is a member of COG1776 family, and part of two component chemotaxis and phototaxis network module in *Halobacterium *NRC-1. It is envisaged that during phototaxis, this protein receives signals from photo transducers HtrI and HtrII and control a flagellar switch [38] enabling the cell to move towards ideal illumination conditions where the light driven proton pump of bacteriorhodopsin converts light energy into chemical energy [39]. The other gene products identified by proteomic analysis were ThiC, ThiD, FumC, ImD2, GapB, TpiA, and PurE, which belong to a category of proteins involved in general metabolic function [40]. In addition, four unannotated gene products of undetermined function were also identified: Vng1807H, Vng0683C, Vng1300H, and Vng6254.

### Survival of *Halobacterium *NRC-1 under heat stress

In order to examine heat shock response in *Halobacterium *NRC-1, differential proteomic strategy was employed; first the conditions for heat shock response were optimized and growth of *Halobacterium *NRC-1 was tested at a wide range of temperatures from optimal (42°C) to lethal (56°C). The data presented in Fig. 2A, show that cells exhibited normal growth up to 49°C, albeit with slower growth rates as compared to the control (42°C). At higher temperatures (>50°C) growth was inhibited and cells appeared white from photobleaching. However, viability at these higher temperatures was largely maintained for several hours and growth resumed after shifting back to 42°C. To further assess the physiological significance of the sublethal but growth inhibitory temperature, we measured survival of *Halobacterium *NRC-1, after pretreatment of cells at sublethal temperature at 49°C for 1 h followed by shifting to a lethal 56°C for up to 6 hours and plating on CM^+ ^plates. A 2.5-fold increase in survival was observed for cells pretreated at 49°C compared to cells that were directly shifted to 56°C (Fig 2B). For the first time it is demonstrated that when growing *Halobacterium *NRC-1 cells are briefly exposed to sublethal temperature they can survive a much more severe temperatures by developing thermotolerance, which is likely to be of ecophysiological relevance to the organism [41]. It seems likely that the increased thermotolerance in heat-shocked cells is ubiquitous in archaea and represents a possible mechanism for survival under thermal stress by induced synthesis of heat shock proteins [42]. As such, a classic heat shock response is observed in *Halobacterium *NRC-1, not surprising since this is a common stress response mechanism adapted and evolved by halophiles inhabiting hypersaline environments.

**Figure 3:**
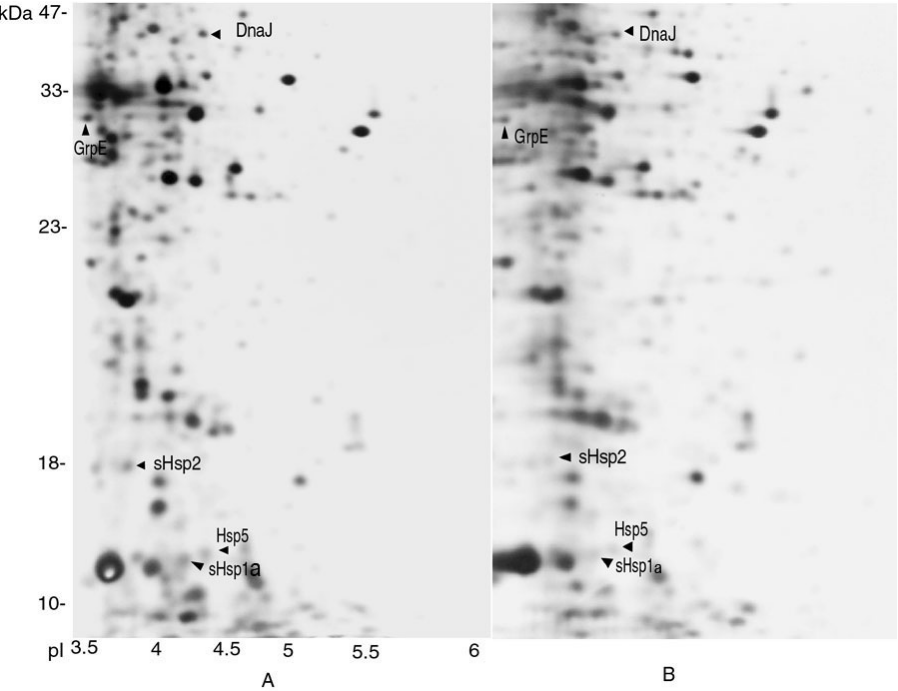
Comparative analysis of 2-D gels from heat shocked (49 °C), and Control (42°C) cells. **A**: Cells shifted and grown at elevated temperature (49 °C); **B**: Cells grown at normal growth temperature (42°C).

**Figure 4:**
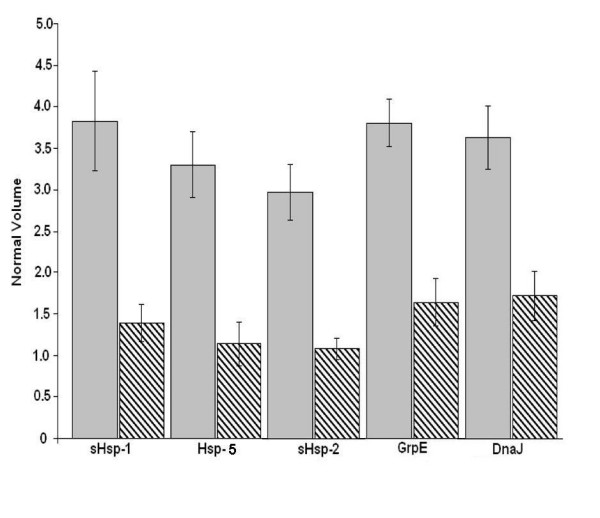
Quantative analysis of heat shock proteins differentially expressed at 42 °C (patterned columns), and 49 °C (shaded columns).

High resolution 2-D PAGE in combination with advanced ImageMaster aided analyses is a powerful tool to study microbial physiology in general. To study the induction and expression of heat shock proteins in *Halobacterium *NRC-1, by employing a differential proteomics approach, exponentially growing cells were subjected to heat shock conditions at 49°C for 8 hrs (generation time of ~6 hrs). Samples containing total cellular proteins were prepared from control and heat shocked cells (equal amounts) and resolved through 2-D gels in triplicate. The gels were digitally imaged after silver staining. The data analyses showed that at least 123 proteins displayed changes in expression when the cells were shifted to elevated temperature. Consequentely, adaptation to high temperature requires substantial change in cellular protein composition [43]. Through an ImageMaster aided comparison of representative sets of gels, we observed that 63 proteins were over expressed under heat shock conditions. At least 37 proteins exhibited a greater than 2-fold increase at 49°C. Conversely in heat shocked cells, the synthesis of 46 proteins was abruptly decreased as compared to control cells growing at 42°C. Based on accurate comparison of representative gels from control, and heat treated samples, it was observed that predicted, and experimental molecular weights, and pIs values of Hsps were closely matched. In the present investigation the precise identification was based on available genomic information, bioinformatic analysis, in combination with experimental data.

The results clearly indicate that when the growing cells are shifted to elevated temperature, there is enhanced synthesis of five heat shock proteins. Among them, three belong to the category of small heat shock proteins (sHsp) and two to the DnaJ family, respectively. Thus, based on bioinformatic analysis and differential proteomic approaches we were able to identify putative molecular chaperones like DnaJ, GrpE, and three small heat shock proteins sHsp-1, Hsp-5, and sHsp-2 in heat shocked cells (Fig. 3A & B; Table 2). In each case, the experimental M_r _and pI values of these putative identified heat shock proteins were close to theoretical values of genomic data. These findings corroborate earlier reports that small heat shock proteins (sHsps) are upregulated significantly under conditions of cellular stress like heat shock [44,45]. Most archaeal genomes contain one or two small heat shock genes, however, in *Halobacterium *NRC-1 genome, there are three copies of *sHsp *[19], which are functional, and their synthesis is up-regulated upon heat stress. However, these proteins have chaperonin properties and are also expressed at basal levels under optimal growth temperatures like the sHsps of other organisms. Thus, it seems likely that sHsps play indispensable roles in the molecular chaperone system of *Halobacterium *NRC-1, and help organism survive under extreme stress conditions. The results indicate that sHsp, DnaJ and GrpE are part of chaperone network which help in refolding of denatured proteins and help normal proteins maintain their native folding state under severe stress [46]. Interestingly, although more than 40 % of all heat shock proteins present in the genome were identified, other expected heat shock proteins, DnaK, Lon, HtrA, and HtpX could not be identified, possibly reflecting the limitation of this approach.

**Table 2:** Bioinformatic analysis of heat shock proteins, identified on 2-DE from thecells grown at normal (42°C), and elevated temperatures (49°C).

HSP name	Predicted MW	Predicted pI	Calculated MW	Calculated pI
DnaJ	41.71	4.36	41.8	4.35
GrpE	23.86	3.95	24.1	3.88
sHsp-1	14.15	4.12	14.15	4.15
sHsp-2	18.53	4.07	18.3	4.04
Hsp-5	14.3	4.25	14.5	4.30

Quantitative analysis of the five putative identified heat shock proteins clearly showed that the three small heat shock proteins sHsp-1, Hsp-5 and sHsp-2 are more than 2-fold induced under heat shock conditions as compared to control (Fig. 4). These three small heat shock proteins predicted in *Halobacterium *genome share ~35 % sequence similarity and belong to Hsp20/alpha crystallin family (PF00011), which is involved in the development of thermotolerance in other systems [47]. Earlier reports have suggested that 16.5 kDa small heat shock proteins from *M. jannaschii *suppress the aggregation of normal proteins in *E. coli *at 100°C [48]. These sHsps are believed to be ATP-independent chaperones that prevent aggregation, and are important in refolding of denatured proteins in combination with other Hsps in the cell. It is also believed that sHsps bind to denatured proteins accumulated under stress conditions, and maintain them in a folding-competent state [49].

There was also more than a 2-fold induction of DnaJ and GrpE in heat shocked cells of *Halobacterium *NRC-1 as compared to control (Fig. 4). Interestingly, in *Halobacterium *NRC-1, DnaJ and GrpE chaperones of bacterial type which form cellular chaperone machinery capable of repairing heat induced protein damage in growing cell [50,51].

## Conclusion

The present report has attempted to optimize conditions to resolve the acidic proteome of *Halobacterium *NRC-1. Acidic proteins tend to precipitate in acidic range and this phenomenon results in poor focusing. Using the modified protocol suggested, it is possible to minimize both vertical and horizontal streaking, allowing proteins to focus in the acidic range. The combination of 2-D gel analysis, LC-MS/MS and MALDI-TOF has enabled the identification of several stress proteins, proteins important for DNA replication and repair, translation regulation, transport, chemotaxis, and housekeeping. For the first time it has been clearly established that *Halobacterium *NRC-1 develop thermotolerance at elevated temperatures through expression of several stress proteins. Furthermore, that expression of stress proteins at under sublethal conditions affords protection from much more severe stresses. By employing differential proteomic approach, five heat shock proteins were identified which were up regulated under temperature stress in *Halobacterium *NRC-1, and allow cells to survive under severe stress.

## Methods

### Preparation of the cell lysate

For protein extraction and cell lysate preparation, *Halobacterium *NRC-1 (ATCC 700922) was grown in batch cultures in CM^+ ^medium with shaking in light. When cultures reached 0.9 OD_600 _the growing cultures were divided and pairs grown in either standard conditions (42°C), or with heat shock (49°C, 8 hours). Triplicate cultures were harvested and cell lysates were prepared. Briefly, cells from 25 ml of culture were collected by centrifugation at 10, 000 × g for 10 min. The cell pellet was resuspended in 2.5 ml of resuspension buffer (5 mM Tris-HCl, pH 8.0, 2 % Tween-20, and 1 mM PMSF, (freshly prepared). The homogenized cell suspension was disrupted using a French Press with three passages at 16000 psi. Unbroken cells and debris were removed by centrifugation at 8000 × g for 10 min at 4°C, and the supernatant containing the soluble fraction of proteins was transferred into a separate tube. Subsequently, the lysate was digested with 100 μg/ml DNase, and 40 μg/ml RNase, for 60 min at 37°C to remove nucleic acids. After rapid cooling to 4°C, the soluble fraction was dialyzed using a 3000 Da cutoff dialysis bag against 5 mM Tris-HCl pH 8.0 for 24 hours at 4°C with at least four changes. Protein concentrations were determined by use of a Bradford dye-based protein assay reagent from Bio-Rad.

### 2-D gel electrophoresis

For 2-DE analysis, 80 μg of dialysed protein extract was mixed with 250 μl of rehydration buffer (8.5 M urea, 2% TWEEN 20, 2 % CHAPS, 0.5 % IPG buffer, pH 3–10, 20 mM DTT and 0.002 % bromophenol blue). After thorough (10 min, 2 min interval) vortexing, the protein sample was centrifuged at 10, 000 × g for 5 min. The cleared protein sample was pipetted into IPG strip holders (IPGphor, Pharmacia Biotech) and incubated with 11 cm IPG ready strips, of the pH range 3–6. The IPG strips were allowed to rehydrate for 16 h at 50 V at 22°C, which enhanced resolution of proteins on the gel. IEF was performed at 500 V for 1 h, 1000 V for 1 h and 8000 V for 2 h for a total of 24000 Vh. After IEF, IPG strips were equilibrated (15 min) in 10 ml Equilibration buffer (50 mM Tris-Cl, pH 8.8, 6 M urea, 30 % glycerol, 2 % SDS, 20 mM DTT and 0.002 % bromophenol blue) followed by re-equilibration (15 min) in the same buffer but containing 20 mM iodoacetamide to minimize the streaking during second dimension electrophoresis. After equilibration, the IPG strip were placed onto a 12.5 % SDS-PAGE gel (18 × 24 cm). The strips were sealed with the help of 0.5 % agarose in electrophoresis buffer. Proteins were electrophoresed at 120 V for 12 h using a Hoefer SE 600 electrophoresis unit. Following electrophoresis, gels were silver stained according to Blum et al [52] and stored in 10 % acetic acid at 4°C.

### Image analyses of 2-D gels

The silver stained gels were scanned using a Kodak EDAS 290 imaging system. Image analysis was performed using the ImageMaster 2D Elite software (Pharmacia Biotech), as described by Krapfenbauer et al 2001 [23]. After spot detection and background subtraction (non spot mode), rigorous editing (automatic and manual), and filtering was performed. Subsequently, gel images were overlaid, and matched, and the quantitative determination of a spots volume was performed (mode: total spot volume normalization). For each analysis, statistical data (from triplicate gels of two independent protein extractions) showed a high level of reproducibility between normalized spot volumes. Normalized volumes of spots from control and experimental gels were exported into Excel (Microsoft) for the calculation of the levels of differential expression.

### Trypsin digestion and gel extraction of peptides

Samples were resolved through 2-D gel electrophoresis using IPG ready strip pH 3–6. Proteins spots were excised from silver stained gels and washed with milli-Q water and twice with 50 % acetonitrile for 15 min. Gel pieces were then washed with a 1:1 solution of 0.1 M NH_4_HCO_3 _and acetonitrile for 15 min. For destaining, gel pieces were incubated in 10 mM DTT/0.1 M NH_4_HCO_3 _for 45 min at 56°C to reduce the protein, followed by incubation in 55 mM iodoacetamide/0.1 M NH_4_HCO_3 _for 30 min at room temperature in the dark for alkylation. Supernatants were discarded and gel pieces were washed with 100 μl NH_4_HCO_3_, followed by two washes (5 min each with vortexing and brief centrifugation) with 100 μl (or enough to cover) of 25 mM NH_4_HCO_3 _in 50% acetonitrile. The gel particles were dehydrated in a Speed Vac (Thermo Savant) to complete dryness and rehydrated with trypsin digestion buffer (50 mM NH_4_HCO_3_, 5 mM CaCl_2_). For trypsin digestion 12.5 ng/μl of porcine trypsin (Promega, Madison, USA) was added in a final volume of 25 μl. Tubes were incubated on ice for 45 min, after which 25 mM NH_4_HCO_3 _was added and tubes were further incubated overnight at 37°C. The supernatant was removed into a clean siliconized tube and extracted twice in 50 % acetonitrile and 5 % formic acid and acetonitrile. The mixture was vortexed 20–30 min and centrifuged. Supernatant was pooled into a separate tube and the volume was reduced to 10 μL using a Speed Vac. Subsequently, the digested mixture was passed through a C18 ZipTip (Millipore). Peptides were eluted into a siliconized tube with 3 μl of 5 % formic acid prior to analysis by MALDI.

### Protein identification by LC-MS/MS and MALDI-TOF

LC-MS/MS analysis was performed by injecting 10 μl sample into the Surveyor HPLC system fitted with BioBasic C-18 packed nanospray tip (New Objective, Woburn, MA) directly coupled to a LCQ Deca XP plus ion-trap mass spectrometer equipped with a nano-LC electrospray ionization source (Thermo Finnigan, San Jose, CA). The spray voltage was 1.70 kV, the capillary temperature was 150°C, and ion-trap collision fragmentation spectra were obtained by collision energies of 35 units. Each full mass spectrum was followed by three MS/MS spectra of the three most intense peaks. The Dynamic Exclusion was enabled. After each sample, an injection of 10 μl of 0.1 % aqueous formic acid was analyzed to ensure proper equilibration of the system.

The 15 raw files were searched against the NCBInr database using SEQUEST (ThermoFinnigan, San Jose, CA), which correlates the experimental tandem mass spectra against theoretical tandem mass spectra from amino acid sequences obtained from the National Center for Biotechnology Information (NCBI) sequence database. Tryptic cleavages at only Lys or Arg and up to two missed internal cleavage sites in a peptide were allowed. The maximal allowed uncertainty in the precursor ion mass was *m*/*z *1.4. Mass spectra were acquired by data-dependent ion selection from a full range as well as discrete and narrow survey scan *m*/*z *ranges to increase the number of identifications

The output files were filtered by Xcorr filter (Xcorr+DeltaCn). The value of XCorr and DeltaCn were Xcorr >2.0 for +2 charged peptides, with partially and fully tryptic ends and DeltaCn >0.1.

MALDI-TOF analysis was performed by reconstituting the dried peptides in 10 μl of 0.1 % TFA and desalted using the C18 ZipTip column from Millipore (Bedford, MA). One μL of concentrated sample was spotted onto the MALDI target plate with 1 μl of 5 mg/ml α-cyano-4-hydroxycinnamic acid in a 1:1 v:v mixture of 50% acetonitrile/0.05% TFA. The sample was allowed to dry for approximately 15–20 min before placing the sample plate to the mass spectrometer for MALDI-MS analysis. The data were collected using the Kratos AXIMA CFR MALDI-TOF (Shimadzu Biotech, USA) in the linear mode. The spectra were internally calibrated using known trypsin autolysis peaks.

The monoisotopic peptide mass fingerprinting data obtained from MALDI-TOF were used to search non-redundant databases [53], using the MASCOT search engine with varying parameter settings [54] (peptide mass tolerance from 0.5 to 1 Da, missed cleavages up to 2). External calibration was performed using Calibration Mixture 2 from the Sequazyme and Peptide Mass Standards Kit (Applied Biosystems, Foster City, CA).

## Abbreviation

2D – two-dimensional gel electrophoresis.

IEF – isoelectric focusing.

PAGE – polyacrylamide gel electrophoresis.

LC-MS/MS – liquid chromatography coupled tandem mass spectrometry.

MALDI-TOF – Matrix assisted laser desorption ionization-time of flight.

HSP – Heat shock proteins.

sHsp – small Heat shock proteins.

## Competing interests

The author(s) declare that they have no competing interests.
